# A trade-off between speckle size and intensity enhancement of a focal point behind a scattering layer

**DOI:** 10.1038/s41598-019-47679-3

**Published:** 2019-08-02

**Authors:** Eitan Edrei, Giuliano Scarcelli

**Affiliations:** Fischell Department of Bioengineering, University of Maryland, 8278 Paint Branch Drive, College Park, MD 20742 USA

**Keywords:** Optics and photonics, Optical physics

## Abstract

Focusing light through highly scattering materials by modifying the phase profile of the illuminating beam has attracted a great deal of attention in the past decade paving the way towards novel applications. Here we report on a tradeoff between two seemingly independent quantities of critical importance in the focusing process: the size of the focal point obtained behind a scattering medium and the maximum achievable intensity of such focal point. We theoretically derive and experimentally demonstrate the practical limits of intensity enhancement of the focal point and relate them to the intrinsic properties of the scattering phenomenon. We demonstrate that the intensity enhancement limitation becomes dominant when the focusing plane gets closer to the scattering layer thus limiting the ability to obtain tight focusing at high contrast, which has direct relevance for the many applications exploring scattering materials as a platform for high resolution focusing and imaging.

## Introduction

Light-based imaging and focusing methods have been historically limited to transparent materials or shallow depths due to multiple light scattering in complex media^1^. In their pioneering work, Vellekoop and Mosk^2^ obtained a “focal point” from a typical random speckle pattern generated behind a highly scattering layer. They achieved this by tailoring the relative phases of light in the scattering medium to constructively interfere at a point of interest. Following this work, in the past decade, several methods have been developed to obtain such intensity enhancement at a focal point either by iteratively modifying the incident beam phase profile with a spatial light modulator (SLM)^2–5^, by directly measuring the optical transmission matrix of the scattering medium^6,7^ or by recording the field fluctuations induced by the medium^8,9^. The various methods designed to enhance a focal point through a scattering layer have attracted a great deal of interest for diverse applications such as deep-tissue focusing^10^, optogenetic modulations^11^, imaging of hidden objects^12^ and high resolution focusing/microscopy^13–16^. The underlying concept shared by these works is that the combination of a scattering medium with spatially-resolved control of the beam phase profile can effectively work as a lens. Several enabling features of such “scattering lens” systems have been described such as super-resolution focusing^17^, versatile focal length and structural compactness^18,19^.

However, although, in relative terms, large intensity enhancements can be obtained at the focal point compared to the average intensity of the speckle pattern, the intensity at the focal point is still typically only several percentages of the incident intensity. To this end, here we report on a tradeoff between the size of the smallest speckle (serving as focal point) that can be obtained behind a thin scattering medium and the intensity of such focal point achieved via wave-front shaping. We present a theoretical derivation and experimental demonstration that as the focal plane gets closer to the thin scattering material leading to smaller speckle sizes, the intensity enhancement of the focal point within this plane is compromised. We show that this intrinsic limit imposes practical constraints on focusing protocols, as it effectively limits the size of a focal point enhanced through a thin scattering layer, and/or sets an upper-bound to the intensity flux delivered to a given location behind a thin scattering medium. In this work we focus on the regime of surface scattering, i.e. scattering originating from a random interface between two materials^20^, the generalization to volume scattering corresponding to thick materials is much more complex and beyond the scope of this study.

## Analytical Model

The intensity enhancement at a single point behind a scattering layer (i.e. the ratio between optimized focus intensity and average background) for a monochromatic coherent light has been previously described as^3^:

1 $${I}_{enhancement}=\gamma N$$

where *N* is the number of controllable degrees of freedom on the phase profile of the illuminating beam and *γ* is an experimental scaling factor (For polychromatic light sources the enhancement is reduced by the number of independent frequency components^21–24^ and for focusing into a general field distribution other factors should be considered^25^). The enhancement in Eq. (1) can be understood intuitively as the result of adjusting the phases (e.g. via an SLM) of *N* independent sub-sources within the beam so that they constructively interfere at a desired location; the pre-factor *γ* depends on several experimental parameters such as the operation mode of the SLM, the sensitivity of the camera to small intensity changes, the noise level throughout the enhancement process and the stability of the scattering medium^26–28^. Here we find that the intensity enhancement is not generally constant when focusing light behind a thin scattering medium and that Eq. (1) represents the upper limit of intensity enhancement that can be reached.

**Figure 1:**
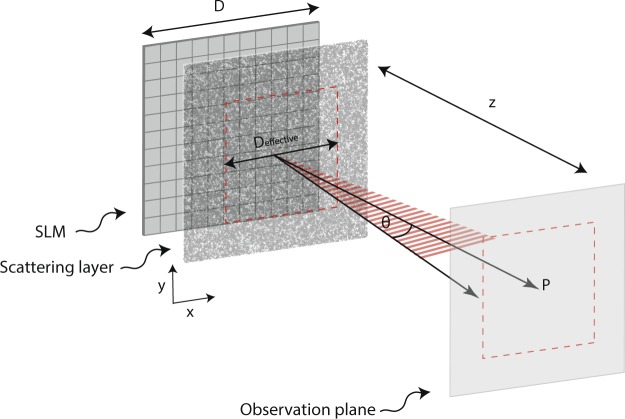
Schematics of a general procedure to form a focal point at location ***P*** in a plane displaced by the axial distant ***z***. The SLM surface is imaged on a scattering layer to spatially control the phase profile of the incident beam.

Let’s consider one typical scenario to achieve an enhanced focal point *P* at a plane located a distance *z* from a scattering layer. To maximize intensity at point *P*; a phase map of linear dimension *D* is projected by an SLM onto the scattering layer and is optimized using a continuous sequential algorithm^29^ (Fig. 1). The configuration we wish to analyze (Fig. 1) in which the SLM plane is conjugated to the scattering layer has been adopted by many studies^13–17^, however, others have implemented a different approach in which the SLM plane is conjugated to the pupil plane of an objective lens placed before the scattering medium^2,6^. In this later approach the wave impinging onto the scattering layer carries a spherical phase curvature, we will address this particular scenario in the discussion section.

Because the scattering process is characterized by a divergence angle *θ*, light diffused by the scattering layer will not be re-directed everywhere; instead, scattering will re-direct light within a cone of angle *θ* centered around the normal to the scattering plane (Fig. 1) and degraded at higher angles. Peripheral locations on the scattering layer for which the central portion of scattering cone is located far away from point *P* will contribute less to the enhancement process. Thus, the illuminated area will not contribute equally to the intensity enhancement. Accordingly, for every experimental scenario, we can define an effective area *A*_*effective*_ which is the area needed to obtain the same enhancement at point *P* when every point equally contributes to the intensity enhancement. The number of SLM pixels which can fit within the effective area, not the actual illuminated area, is the quantity which will determine the enhancement efficiency.

To quantify *A*_*effective*_, we model the illumination plane as a collection of scattering point sources and assign a weight factor to each point based on their effective contribution to the intensity of focal point *P*. Specifically, let us assume that each scattering point source generates a beam with a transverse gaussian profile of width determined by the scattering angle *θ* and by the propagation distance *z* (i.e. with standard deviation *z*⋅*tan*(*θ*)). The effective area is the weighted integral of all the scattering point sources:

2 $${A}_{effective}={\int }_{0}^{D}\,{\int }_{0}^{D}\,{e}^{\frac{-({x}^{2}+{y}^{2})}{2{(z\cdot tan\theta )}^{2}}}dxdy$$

where *x*, *y* are the spatial coordinates in the plane of the scattering layer (i.e: *z* = 0). In the limit of large distances from the scattering layer, i.e. for *z* → ∞, the integration of Eq. (2) yields *A*_*effective*_ = *D*^2^, i.e. all SLM pixels equally contribute to the optimization process. This is the ideal situation described by Eq. 1.

For finite distances from the scattering layer, the integration of Eq. (2) can be solved analytically by substitution, $\tilde{x}\,(\tilde{y})=x(y)\cdot \frac{1}{\sqrt{2}\cdot ztan(\theta )}$, to give:

3 $${A}_{effective}=\frac{\pi }{2}\cdot {(z\cdot \tan \theta )}^{2}\cdot {[{\rm{erf}}(\frac{D}{\sqrt{2}\cdot z\cdot \tan (\theta )})]}^{2}$$

Thus, only the number of SLM pixels contained within the effective illumination area will contribute to the enhancement. This leads to the general form of Eq. (1):

4 $$\begin{array}{rcl}{I}_{enhancement} & = & \gamma N\cdot \frac{{A}_{effective}}{A}\\  & = & \gamma N\cdot \frac{\pi }{2{D}^{2}}\cdot {(z\cdot \tan \theta )}^{2}\cdot {[{\rm{erf}}(\frac{D}{\sqrt{2}\cdot z\cdot \tan (\theta )})]}^{2}\end{array}$$

In summary, the ideal enhancement would be reached in perfectly isotropic scattering conditions where the light is distributed equally over a solid angle of 2π after the scattering medium. Instead, even though the intensity distribution of the SLM pattern projected onto the scattering layer is uniform, the finite divergence angle of scattering phenomenon decreases the contribution of peripheral locations. The intensity enhancement can be interpreted as arising from a radially degrading intensity distribution, which directly affects both the focal intensity and the effective numerical aperture.

We can also analyze the behavior of the intensity enhancement as a function of the unitless parameter:

5 $$\begin{array}{rcl}U & = & \frac{D}{\sqrt{2}\cdot z\cdot \,\tan \,(\theta )}:\\ {I}_{enhancement} & = & \gamma N\cdot \frac{\pi }{4}\cdot {(\frac{1}{U})}^{2}\cdot {[erf(U)]}^{2}\end{array}$$

We note two limits: $\mathop{\mathrm{lim}}\limits_{U\to \infty }[\frac{\pi }{4}\cdot {(\frac{1}{U})}^{2}\cdot {[{\rm{erf}}(U)]}^{2}]=0,\,\,\mathop{\mathrm{lim}}\limits_{U\to 0}[\frac{\pi }{4}\cdot {(\frac{1}{U})}^{2}\cdot {[{\rm{erf}}(U)]}^{2}]=1.$ The first limit occurs for focal planes very close to the scattering layer, i.e. *z* ≪ *D*. Under these circumstances, the effective illumination area vanishes and the enhancement approaches zero (our derivation, though, does not consider the evanescent field and is restricted to *z* > *λ*). The second limit refers to when the focal plane is far from the scattering layer. In this case, the effective illumination area is the entire illumination area, and the enhancement approaches the optimal *γN* value. Interestingly, this limit can be expressed as: $\frac{D}{\sqrt{2}\cdot z\cdot \,\tan (\theta )}\ll 1$ and reduces to

6 $$z\gg \frac{d\cdot D}{\lambda }$$

where d is the average linear distance between scattering particles (i.e. the correlation length) and we approximated the scattering angle as $tan(\theta )\approx \theta \approx \frac{\lambda }{d}$^30^ (valid under the condition *d* > *λ*). This limit exactly coincides with the ‘far-field’ condition for partial coherent light: $z\gg \frac{{L}_{c}\cdot D}{\lambda }$ derived from the propagation of mutual coherence (generalized Van Cittert- Zernike theorem)^20,31,32^, where *L*_*c*_ is the correlation length right after the scattering material, which was shown to approach d^30^. Interestingly, while the focal length of scattering lenses has been so far assumed to be entirely variable^14,33^, here we find that a ‘far-field’ condition needs to be met for optimal focusing.

## Results

**Figure 2:**
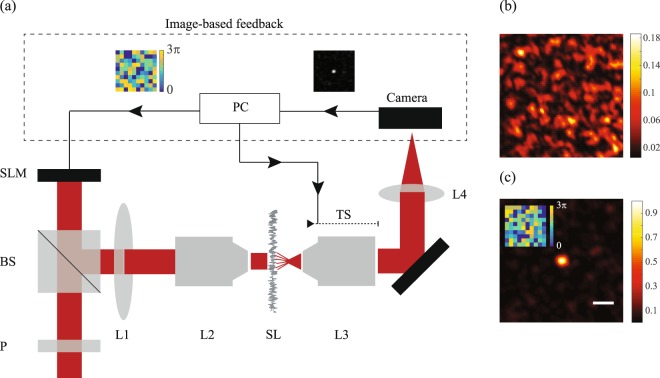
(**a**) Experimental setup: the SLM surface (divided into 100 macro-pixels) was imaged via a 4-f imaging system (L1, L2) onto the scattering layer. A plane behind the scattering layer was imaged onto the camera, and a location within the imaged plane was enhanced by applying a feedback loop to vary the phase map of the SLM. The imaged plane was selected by adjusting the translational stage of L3. (**b**) Intensity distribution pattern before the enhancement process. (**c**) Intensity distribution after the enhancement process (scale bar = 5 µm). The inset shows the final SLM phase map to which the algorithm converged.

To experimentally verify our theoretical predictions, we built the setup in Fig. 2(a). A polarized expanded laser beam of *λ* = 660 *nm* (LaserQuantum) was reflected off the surface of phase-only SLM (Hamamatsu X10468-01). The SLM plane was then imaged on a 600-grit diffuser (Thorlabs) which served as scattering medium. To vary the illumination area, we used a de-magnifying 4-f imaging system, with L1 of focal length 400 mm and L2 of variable focal length (45 mm to 3 mm). An infinitely corrected imaging system was used after the scattering layer to record a plane of interest (L3 = 0.75 NA, 20X, L4 = 200 mm). The distance between the scattering medium and the observation plane (i.e. the plane of enhancement) was selected by adjusting the translational stage of L3. To enhance a single point beyond the scattering layer, the SLM was divided into 100 macro-pixels, and each pixel was varied individually from 0 to 3*π* to determine the optimal phase configuration by using the recorded pattern on the camera as feedback. This process was repeated for all pixels twice, for a total ~20 *min* per enhancement process.

First, we verified our ability to focus light through a scattering material consistently with traditional protocols. We imaged a plane located 1.6 mm after the scattering layer (corresponding to *z* ~ 3.2 *D*) and as expected obtained a speckle pattern, shown in Fig. 2(b). We selected a central location in the pattern, performed the sequence to enhance the intensity recorded at that location. After two iterations of every pixel, we arrived at the final intensity distribution presented in Fig. 2(c). The number of degrees of freedom controlled by the SLM is orders of magnitude smaller than those needed to perfectly correct for the variations of the scattering medium and thus, the optimization of relative phases re-directs a small portion of the light energy to form a high contrast focal point. We reached an enhancement of 32 corresponding to *γ* ~ 0.3, consistent with previously reported values^3,28^.

**Figure 3:**
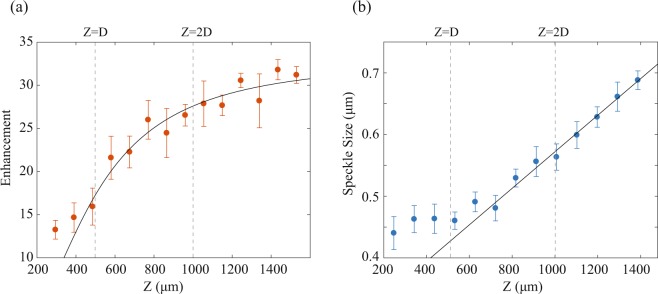
(**a**) Intensity enhancement at different focusing planes of varying distance from the scattering layer. Experimental data (orange dots) are well fit by Eq. (4) (black line). The optimization algorithm was performed >10 times for each data point and results below the median were discarded to eliminate artifacts due to mechanical vibrations or material decorrelation. (**b**) Speckle size at different focusing planes of varying distance from the scattering layer. Experimental data are calculated by first performing an autocorrelation of the speckle pattern then measuring the width at HWHM of the peak and dividing it by $\sqrt{2}$ to account for the autocorrelation broadening^42^ (blue dots). A linear fit (black line) fits well the data after the critical distance.

Next, we directly demonstrated the prediction of Eq. (4). We de-magnified the SLM onto the scattering layer to an area of linear dimension *D* = 500 *μm*, and executed the enhancement protocol at planes of different distances from the scattering layer. Figure 3(a) shows the intensity enhancement as the selected focal plane gets closer to the scattering layer (orange dots). The black line is a fit to the experimental data using Eq. (4) and keeping tan(*θ*) as a free parameter. The intensity enhancement is not constant as Eq. (1) would predict but increases with the distance from the scattering layer, in agreement with the theoretical prediction of Eq. (4).

Figure 3(b) shows the corresponding average speckle size obtained at various distances from the scattering layer: as expected the speckle size is constant until a critical distance (${z}_{c}\approx \frac{d\cdot D}{\lambda }$)^20,31^ and then scales up linearly. Interestingly, this effect can also be explained using the effective area concept: before the “far-field” condition of Eq. 6, the effective area contributing to the constructive interference proportionally decreases and thus prevents further reduction of the speckle size^20^. Figure 3(a,b) are consistent with each other: using the value for *θ* obtained from the fit of Fig. 3(a), the critical axial location for linear speckle growth is *z*_*c*_ ≈ 1.65*D*. Since in this experiment *D* = 500 *μm* this yields *z*_*c*_ ≈ 800 *μm* which corresponds to the transition to linear speckle growth in Fig. 3(b).

**Figure 4:**
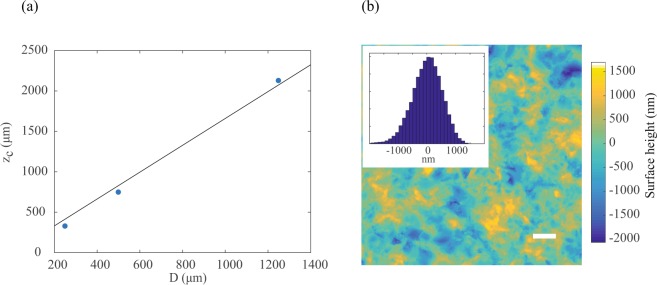
(**a**) Critical distance ***z***_***c***_, evaluated as the axial location corresponding to 73% of the maximal intensity enhancement, vs the illumination linear dimension ***D*** (blue dots). The black line is a linear fit to the data through the origin of the coordinates. (**b**) AFM surface height map of the scattering layer (scale bar = 10 ***μm***). The inset shows the distribution of surface heights.

To prove the universality of our findings, we repeated the experiments in Fig. 3 for three different sizes of illumination (*D* = 250 µm, 500 µm, 1250 µm) of the scattering layer. To compare the results, we considered that Eq. (4) reduces to *I*_*enhancement*_ ≈ 0.73*γN* at the critical distance *z* = *z*_*c*_(under the approximation $tan(\theta )\approx \theta \approx \frac{\lambda }{d}$)^23^. Therefore, for each illumination size, we plot the value of *z* corresponding to 73% enhancement of the maximal value, which should correspond to the critical distance *z*_*c*_. The results are presented in Fig. 4(a).

The correlation between *D* and *z*_*c*_ is expected to be governed only by the divergence angle: $\theta  \sim \frac{\lambda }{d}$ and is therefore constant for any illumination size. Indeed, the data from the three illumination sizes are well described by a linear fit. From the slope of the fit, we extracted the scattering divergence angle $\theta  \sim \frac{\lambda }{d}=0.6$, corresponding to $d=\frac{\lambda }{0.6}=1100\,nm$. We confirmed the estimate of the scattering scale *d* by mapping the height of the diffuser with an Atomic Force Microscope (AFM) as shown in Fig. 4(b). From the AFM measurement the FWHM of the variation distribution is ~1200 *nm*,in good agreement with our calculated value (we obtained similar results by measuring the spatial variations across the diffuser). In biological tissues the scattering angle is typically smaller with forward scattering being the preferred direction^34,35^.

## Discussion

In summary, in this work we derived and verified the intrinsic limits of intensity enhancement that can be reached when focusing light through a thin scattering material at different axial locations. Our study is confined to scenarios of highly scattering materials in which the number of controllable elements (i.e. SLM pixels) is much lower than the one needed to produce a perfect complementary phase map. Thus, the divergence angle is dictated by the scattering material rather than any optical element placed within the illumination path. In our study we analyzed only the scenario in which the unmodified wave front propagating from the SLM to the scattering medium is a plane wave. If instead, a converging spherical wave is generated prior to the scattering medium, the result will depend on the extent to which the incoming phase is preserved throughout the scattering process. As more scattering events occur, any memory of the original incoming wave-front phase is lost and does not play a role in the focal enhancement process. Yet, for thin mildly scattering samples further studies are required to determine the potential effect of spherical convergence on the enhancement of a focal point beyond the scattering medium.

This work has direct relevance for the many studies that use scattering materials as a platform for high resolution microscopy/focusing^13–15,36^. Our analysis can determine the maximum contrast achievable when high-resolution or super-resolution is attempted with scattering lenses. Optimal enhancement is achieved by imaging as many SLM pixels as possible into the effective illumination area calculated here; thus, in practice, the ultimate enhancement limit is reached when the SLM pixels are de-magnified to the smallest size allowed by the imaging system that projects the SLM map onto the scattering material. In the evanescent-wave regime (*z* ~ 100 *nm*) sub-wavelength resolutions can be reached^37^; but, the effect of the divergence angle will need to be considered. The effective illumination area in the evanescent regime is expected to be reduced to several microns, which will limit the intensity enhancement; this could explain why experimentally the enhancement was found to be far from optimal in this regime^17^. For practical applications, it will be important to establish how much intensity enhancement is required to achieve sufficient contrast for specific purposes, such as fluorescence excitation, neural activity modulations or label-free imaging. This will ultimately determine how close the focusing plane can be set and how high resolution can be achieved. The phenomenon we describe here may also be applicable to the emerging field of focusing and imaging through multimode fibers^38–40^ and fiber bundles^41^ as the enhancement capabilities at close proximity to the fiber outlet is expected to decrease.
